# Editorial: Digitizing frozen earth—revealing microbial diversity and physiology in the cryobiosphere through “omics” tools, volume II

**DOI:** 10.3389/fmicb.2022.1013398

**Published:** 2022-09-30

**Authors:** Anne D. Jungblut, David Velazquez, Samuel Cirés, Julia Kleinteich, Krishnan Kottekkatu Padinchati, Birgit Sattler, Jérôme Comte

**Affiliations:** ^1^Life Sciences Department, Natural History Museum, London, United Kingdom; ^2^Biology Department, Universidad Autonoma de Madrid, Madrid, Spain; ^3^German Federal Institute of Hydrology, Koblenz, Germany; ^4^Arctic Ecology and Biogeochemistry Division, National Institute for Polar and Ocean Research, Vasco da Gama, India; ^5^Department of Ecology, University of Innsbruck, Innsbruck, Austria; ^6^Austrian Polar Research Institute, Vienna, Austria; ^7^Institut National de la Recherche Scientifique, Centre Eau Terre Environnement, Quebec City, QC, Canada; ^8^Centre d'Études Nordiques (CEN), Université Laval, Quebec City, QC, Canada

**Keywords:** Arctic, Antarctica, alpine environments, polar microbiology, high throughput sequencing

Terrestrial, marine and freshwater habitats of the three poles (Arctic, Antarctica, and high-altitude regions) have striking similarities in their environmental properties. The harsh conditions permit the survival of a unique selection of (micro)organisms. Global change is unfolding across polar and alpine regions without precedents in human history (IPCC, 2022). The current human development paradigm has important environmental consequences and all forms of life thriving in the cryosphere face pressures with consequences beyond local scales. Microbes ranging from Bacteria, Archaea, and Eukaryotes (i.e., algae, protists, and fungi) are not only able to thrive in permanently cold environment but also are an important component of foodwebs and players in aquatic and terrestrial ecosystems function, microclimate regimes and any possible response of the polar and alpine regions to anthropogenic warming (Cavicchioli et al., 2019; Tiedje et al., 2022). They are the first responders to environmental changes and act as game-changers of key ecological process kinetics. Microbial ecology research today faces the challenges of still quite complex and unresolved issues to foster its understanding of the natural world.

Effects of global warming on the physical, chemical, ecological structure, and function and biodiversity of polar and alpine ecosystems are not well-understood and there are many opinions on how microbes might respond and adapt to environmental stress and long term changes in environmental conditions in freshwater, ice and soil environments (Guan et al., 2017). At the same time climatic driven environmental change and an increasing number of extreme events are being observed. In order to be able to develop a better understanding of the potential effect of climatic perturbations on ecosystems structure and function at the Three Poles, it requires more details on the ecological drivers of community assemblies and resolution on seasonal and longer time scales that microbial assemblages might respond to environmental change. This might also help to identify sentinel taxa or habitats that could act as indicators of change including ice-based ecosystems as part of the Last Ice Area. The increasing use of “omics” techniques to disentangle microbial diversity and ecosystem function in the cryosphere is now offering new avenues to investigate the response of polar and alpine ecosystems microbial communities to global warming (Abatenh et al., 2018; Edwards et al., 2020).

The aim of this Research Topic “*Digitizing frozen earth—Revealing microbial diversity and physiology in the Cryobiosphere through “Omics” tools, volume II*” is to provide readers with a selection of studies that are using the latest “omics” and multidisciplinary approaches as well as go beyond the description of microbial communities to evaluating mechanistic processes that shape environmental microbial assemblages. Along this Editorial, we present and highlight with concrete examples how this walkthrough—from boots to bytes—is carried out by the scientific community to overcome the hurdles from samples to integration of large ecosystem processes. Contributions include original research studies on understudied taxonomic groups such as protists, fungi, and uncultured candidate bacterial groups as well as evaluation of mechanistic processes driving community assembly across habitats and seasons in polar and alpine environments based on field and laboratory studies. Research featured in this Research Topic also investigates the response of microbial communities to environmental factors and changing conditions along extended temporal scales. The Research Topic brings together studies from the Three Poles; covering Arctic, Antarctic and high altitude environments, to allow a comparison and perspective on the similarities and differences of these permanently cold but geographically separated environments.

The Research Topic includes contributions that demonstrate the presence of key candidate bacteria groups in Antarctic lakes and their potential physiology and contribution to biogeochemical processes based on high-throughput “omics” techniques. Williams, Allen, Berengut et al. outlines a characterization of novel *Cloacimonadota* and *Omnitrophota* from Ace Lake as well as Candidatus *Organicella extenuata*, a Verrucomicrobial endosymbiont with a reduced genome, from Organic Lake, Vestfold Hills in Antarctica (Williams, Allen, Ivanova et al.). While bacteria have been covered by many 16S rRNA gene high throughput sequencing surveys and dominating environmental metagenomic studies, there is still a surprising paucity of data for protists and fungi. Millar et al. showed that 18S rRNA gene based protists and fungi assemblages in supraglacial cryoconite include potential grazers, predators, photoautotrophs that show distinct communities in the Arctic and Antarctic similar to prokaryotic communities. The study by Ilicic et al. focused on microbial eukaryotes and fungi in shallow marine coastal zones in Potters Cove (South Shetland Islands, Western Antarctic Peninsula), and their assessment of fungi suggests that Ascomycota and Chytridiomycota fungi are the most abundant taxa in these benthic microphytobenthic habitats including putative fungal parasites. Furthermore, the review by Gilbertson et al. focuses on polar marine diatoms and evolution and adaptation mechanisms based Omics approaches. All three contributions highlight that there is a need for a more comprehensive assessment of these taxonomic groups.

As the climate continues to warm, the cryospheric environments will experience drastic changes in their structure: ice thickness reduces and aquatic ecosystems become more open, the atmosphere increasingly exchanges with the terrestrial environments, and the active layer of soil is deepening as permafrost thaws. These changes occur at different temporal and spatial scales but collectively represent a strong environmental filter for local microbial communities that have to either respond and adjust to these new conditions or new niches are now available for previously rare taxa or originating from the surrounding environment (Leibold et al., 2004; Hanson et al., 2012; Lindström and Langenheder, 2012). The contributions of this Research Topic explore the mechanisms for selection and habitat filtering of microbial communities and lead to local variation in assemblages in permanently cold environments. The studies also highlight the microbiomes are usually influenced by multiple factors and the difficulty of untangling them. For example, Fillinger et al. describes that bacteria cell number and virus-like particles are potentially influenced by both spatial and temporal drivers in the European Alps. Rapp et al. also found distinct difference in composition and the genomic traits in communities from Arctic seawater-derived subzero hypersaline brines from 1st year ice (near Barrow Sea Ice Balance Site, Alaska) and ancient cryopeg (Barrow Permafrost Tunnel, Alaska) in permafrost.

Marois et al. suggested that local habitat filtering shapes the planktonic microbial community structure in four chemically and physically distinct lakes on northern Ellesmere Island, Canadian Arctic Archipelago that is part of the coastal margin zone of the Last Ice Area. While Blais et al. investigated river bacterial communities along dissolved organic carbon (DOC) and salinity gradients in the Great Whale River Subarctic, Canada, and found that a core microbiome in subarctic rivers and a coastal plume contained generalists but also taxa with a more limited distribution. However, many studies to date cover mostly the evaluation of the relationship between microbial communities and environmental variables during the short duration of the polar summer due to logistical constraints, and there is therefore a lack of field studies during the remaining and majority of the year between autumn, winter and spring. Extending studies across seasons not only will provide a more complete understanding, but they will also provide valuable data in context of climate change research as it has been predicted that warming in the polar regions will lead to changes in the seasonality. In this context, a comparison between planktonic 18S rRNA gene communities in lakes in Ekaluktutiak (Cambridge Bay, southern Victoria Island, Nunavut, Canada) found differences in the mixotrophic and heterotrophic core communities in open water and under the ice conditions (Potvin et al.).

Environmental gradients and chronosequences provide opportunities for natural laboratories to evaluate the response of microbial communities to a change of conditions across space and time which is explored in several studies on soil ecosystems. The space-for-time substitution was applied to study the response of soil microbial communities to water availability and geochemical soil properties in the McMurdo Dry Valleys as the polar deserts are predicted to experience drastic changes in water availability under current climate change predictions (Monteiro et al.). Liu et al. evaluated the changes of pioneer prokaryotic communities in rhizosphere and bulk soils along the high-elevation glacier retreat chronosequence, the northern Himalayas, Tibetan Plateau, and found that prokaryotic community composition during colonization and succession are shaped by a combination of plants, habitat and duration since glacier retreat. In the permafrost environment, Scheel et al. was able to do first prokaryotic 16S rRNA and fungal ITS2 gene regions sequencing study of an abrupt permafrost erosion microbiome in Northeast Greenland, where a thermal erosion gully collapsed, leading to the thawing of 26,500-year-old permafrost material. Finally, a laboratory-based microcosm experiment was presented by Malard and Pearce to evaluate the colonization of snow derived bacteria deposited onto Arctic soils, demonstrating that potentially successful colonizations of soil by invading bacteria could be influenced by local soil properties rather than by ecosystem disturbances.

In summary, with ongoing climate warming and rapid alteration of the cryosphere at the Three Poles, a mechanistic understanding of the response of microbial communities is greatly needed. The broad-range of articles presented here deepen our current understanding and knowledge of the mechanistic processes that shape environmental microbial assemblages. This Research Topic of studies identified many challenges and questions that yet remain to be addressed. We are hopeful that this Research Topic will stimulate discussions and paved the way for future avenues of research.

## Author contributions

AJ, JC, BS, DV, and SC conceived of the manuscript. JK and KK contributed and commented on the article. All authors contributed to the article and approved the submitted version.

## Funding

We thank Sentinel North (CFREF) and ArcticNet (NCE) for funding support for the T-MOSAiC workshop. Natural Sciences and Engineering Research Council of Canada (NSERC, #RGPIN-2020-06874). DV and SC were supported by CAM-UAM funds (ref.: SI3-PJI-2021-00461).

## Conflict of interest

The authors declare that the research was conducted in the absence of any commercial or financial relationships that could be construed as a potential conflict of interest.

## Publisher's note

All claims expressed in this article are solely those of the authors and do not necessarily represent those of their affiliated organizations, or those of the publisher, the editors and the reviewers. Any product that may be evaluated in this article, or claim that may be made by its manufacturer, is not guaranteed or endorsed by the publisher.
